# Bio-Mimic Optimization Strategies in Wireless Sensor Networks: A Survey

**DOI:** 10.3390/s140100299

**Published:** 2013-12-24

**Authors:** Md. Akhtaruzzaman Adnan, Mohammd Abdur Razzaque, Ishtiaque Ahmed, Ismail Fauzi Isnin

**Affiliations:** 1 Faculty of Computing, Universiti Teknologi Malaysia, 81310 Skudai, Johor, Malaysia; E-Mails: marazzaque@utm.my (M.A.R.); fauzi@cs.utm.my (I.F.I.); 2 Faculty of Civil Engineering, Universiti Teknologi Malaysia, 81310 Skudai, Johor, Malaysia; E-Mail: ishtiaque@utm.my

**Keywords:** wireless sensor networks, optimization, bio-mimetic algorithms, particle swarm optimization, ant colony optimization, genetic algorithm

## Abstract

For the past 20 years, many authors have focused their investigations on wireless sensor networks. Various issues related to wireless sensor networks such as energy minimization (optimization), compression schemes, self-organizing network algorithms, routing protocols, quality of service management, security, energy harvesting, *etc.*, have been extensively explored. The three most important issues among these are energy efficiency, quality of service and security management. To get the best possible results in one or more of these issues in wireless sensor networks optimization is necessary. Furthermore, in number of applications (e.g., body area sensor networks, vehicular *ad hoc* networks) these issues might conflict and require a trade-off amongst them. Due to the high energy consumption and data processing requirements, the use of classical algorithms has historically been disregarded. In this context contemporary researchers started using bio-mimetic strategy-based optimization techniques in the field of wireless sensor networks. These techniques are diverse and involve many different optimization algorithms. As far as we know, most existing works tend to focus only on optimization of one specific issue of the three mentioned above. It is high time that these individual efforts are put into perspective and a more holistic view is taken. In this paper we take a step in that direction by presenting a survey of the literature in the area of wireless sensor network optimization concentrating especially on the three most widely used bio-mimetic algorithms, namely, particle swarm optimization, ant colony optimization and genetic algorithm. In addition, to stimulate new research and development interests in this field, open research issues, challenges and future research directions are highlighted.

## 1. Introduction

With the advancements in Micro-Electro-Mechanical Systems (MEMS) technology, wireless sensor networks (WSNs) have gained worldwide attention in recent years. A large number of applications including medical care, habitat monitoring, precision agriculture, military target tracking and surveillance, natural disaster relief, hazardous environment exploration and monitoring are all using this technology. Wireless Sensor Networks (WSNs) are critically resource-constrained by their limited power supply, memory, processing performance and communication bandwidth [1]. Due to their limited power supply, energy consumption is a key issue in the design of protocols and algorithms for WSNs. Hence, most existing works (e.g., clustering, lifetime prolonging) in the WSN area are dealing with energy efficiency. Typically, this energy consumption minimization or efficiency is not a trivial task, as in most cases number of conflicting issues need to be considered (e.g., lifetime, coverage). Optimization is very helpful in making the appropriate tradeoffs between these conflicting issues to get the best possible results [2].

Like energy efficiency, Quality of Service (QoS) is necessary in a number of WSN applications such as Body Area Networks (BANs), Vehicular *ad hoc* Networks (VANETs), military target tracking and surveillance, *etc.* Obtaining QoS in these highly resource-constrained networks is not an easy task. In a number of cases, QoS metrics or parameters might even conflict with themselves. For example, in almost all medical applications, timeliness or on time delivery is compulsory, but that may conflict with energy efficiency (considering it as a QoS parameter), so the use of optimization is necessary in all these conflicting QoS scenarios. Like QoS and energy efficiency, security is another key concern for a number of WSN applications. Potential security measures could include a method of assuring that the packet/data was generated by a trusted source (sensors), as well as a method of assuring that the packet/data was not tampered with or altered after it was generated. Security may conflict with energy efficiency and QoS in a number of WSN applications. For instance, to ensure security, the use of encryption algorithms is very common, but this may lead to longer processing times that conflict with timeliness (QoS) of real-time data delivery, and the energy efficiency of WSN applications. Hence optimization is necessary to make a trade-off between these three.

Unfortunately, most conventional or classical optimization algorithms like the Hessian matrix-based methods and gradient-based methods [3,4] are not suitable for WSNs. In conventional optimization approaches, the methods need to comply with the structure of the objective function which is to be solved [2], but sometimes the derivative of the objective function cannot be calculated. Therefore the optimal result becomes hard to find using classical algorithms [5]. For the last two decades bio-mimetic strategies have been widely used to solve these issues as they can solve non-differential nonlinear objective functions which are really hard to find using classical algorithms.

Thus, bio-mimetic optimization algorithms with some degree (low or medium) of computational complexity are worth exploring. Conventional or classical optimization algorithms are power hungry approaches. They must be restructured to reduce code size and dynamic memory usage due to the limited memory capacity of WSN nodes—typically less than 50 KB for code memory and even less for data memory. Recently, researchers have addressed these challenges by adopting bio-mimetic optimization strategies along with conventional techniques. There exists a diverse range of bio-mimetic or metaheuristic algorithms for optimization in wireless sensor networks including Particle Swarm Optimization (PSO), Genetic Algorithm (GA), Ant Colony Optimization (ACO), *etc.* In fact, optimization algorithms are far more diverse than the types of optimization, but the right choice of an optimization algorithm can be crucially important in finding the right solutions for a given optimization scenario.

Optimization, especially bio-mimetic strategy-based optimization in WSNs, is a very active research area. Papers published in this area are highly diverse in their approaches and implementations. To the authors' knowledge, there is no article which provides survey of the area. However, some work has been done addressing the various issues individually (e.g., energy efficiency, QoS or security) and they tend to overlook the whole scenario of collective optimization approach which encompasses these two or three WSN issues. In [6], an extensive survey was done on WSNs taking into account the topic of overall computational intelligence, but with some focus on bio-mimetic strategies. The more recent survey [7] narrowed down its focus to an ant colony optimization (ACO)-based approach to solve several issues in WSNs. Moreover, in [8] the authors discussed a protocol based on ACO, and two fundamental parameters, QoS and reputation are used. Both works exclude other popular techniques like PSO and GA. In [9], some issues of WSNs have been addressed using only PSO. A number of papers have reported works on energy efficient clustering [10–13] and prolonging network lifetime [14] in WSNs using PSO.

Considering these points, we feel that now is an appropriate time to put recent works into perspective and take a holistic view of the field. This article takes a step in that direction by presenting a survey of the literature in the area of bio-mimetic optimization strategies in WSNs focusing on current, ‘state-of-the-art’ research. This paper aims to present a comprehensive overview of optimization techniques especially used in energy minimization, ensuring security, and managing QoS in WSN applications. Finally, this work points out open research challenges and recommends future research directions.

Section 2 presents a brief overview on optimization and Section 3 presents the rationale for optimization in WSN in details. Section 4 provides an overview of existing approaches of bio-mimetic optimizations including hybrid approaches in WSNs. Open research challenges and suggestions for future research directions are presented in Section 5. Finally Section 6 concludes the work and points to areas of potential future work.

## 2. Optimization Strategies

### 2.1. What is Optimization?

Optimization is a term that covers almost all sectors of human life and work; from scheduling of airline routes to business and finance, and from wireless routing to engineering design. In fact, almost all research activities in computer science and engineering involve a certain amount of modeling, data analysis, computer simulations, and optimization [15]. In a word, it is an applied science that tries to obtain the related parameter values which facilitate an objective function to produce some minimum or maximum value [2]. In the real world, resources are limited, time and money are always less than required, so optimization is far more important in practice [16–18].

**Figure 1.**
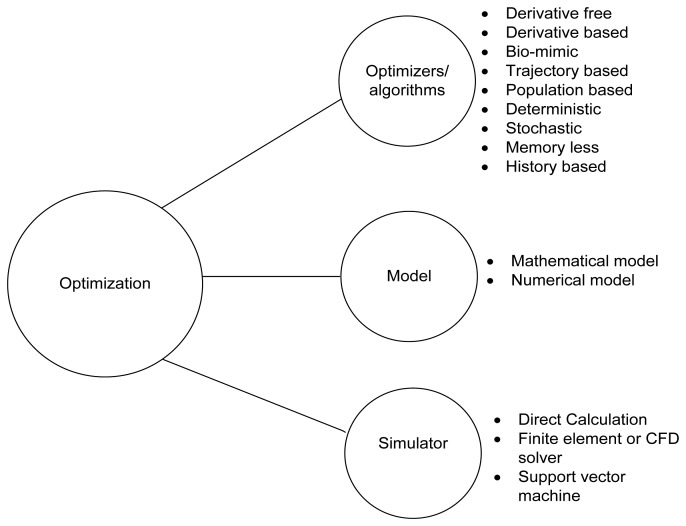
A simple optimization process.

A typical optimization process consists of three components: model, optimizer and simulator (see Figure 1). The representation of the physical problem is done by using mathematical equations which can be converted into a numerical model. The formulation of a simple optimization problem can be done in many ways [15].

For instance, the most popular way to do the formulation is to write a nonlinear optimization problem as:

(1) $$\text{minimize}\;f_{i}\left(x\right),\left(i=1,2,\ldots ,M\right),$$

subject to the constraints:

(2) $$\begin{array}{cc} h_{j}\left(x\right), & \left(j=1,2,\ldots ,J\right), \end{array}$$

(3) $$g_{k}\left(x\right),<0\left(k=1,2,\ldots ,K\right),$$

where *f_i_*, *h_j_* and *g_k_* are nonlinear functions. Here the design vector *x* = (*x*_1_, *x*_2_, …) can be continuous, discrete or mixed in n-dimension [15]. The function *f_i_* is called objective function (cost function). Here when *M* is 1, it is a single objective function. But when *M* > 1, the optimization is multi objective [19]. It is possible to combine different objectives into a single objective and in some cases it is a useful approach. It can be noted that the problem we formulated here is a minimization problem. The maximization problem can be written by simply substituting *f_i_*(*x*) by −*f_i_*(*x*).

When *K* = 0, the optimization turns out to be an equality constrained problem, as we have only the equality constraints left. Equality *h*(*x*) = 0 can be expressed as two inequalities: *h*(*x*) ≤ 0 and −*h*(*x*) ≤ 0. It is important to mention that a number of formulations in the optimization literature use constraints with only inequalities.

We are dealing with nonlinear constrained problems when all the functions are nonlinear. In some particular circumstances when *f_i_*, *h_j_*, and *g_k_* are linear, the problem itself becomes linear. In this case we can apply the broadly used linear programming methods. If the problem is of mixed type, meaning some design variables take discrete values, while other variables take real continuous values, it is often complicated to solve them, especially when the optimization problem is large-scale.

### 2.2. Optimization Algorithms

Choosing a proper algorithm or optimizer is an important step of any optimization. An efficient optimizer is vital to make sure that an optimal solution is reached. There is no single algorithm which is suitable for all problems. There exist a number of optimization algorithms including derivative-based algorithms (also known as gradient-based algorithms), derivative-free algorithms and bio-mimetic algorithms. The first two algorithm types are known as classical optimization methods. They are generally either Hessian matrix-based methods or gradient-based methods [3,4], whereas most of the bio-mimetic algorithms use pattern matrix-based methods which give random solutions to the related problems. This method enables the information exchange between the patterns and results in significant improvement.

#### 2.2.1. Derivative-Based Algorithms

This type of algorithms uses the information of the derivative. As they have proved their competence as local search algorithms, they are widely used in many scientific applications and in discrete modeling [20,21]. One disadvantage of this method is that, if the problem of interest is not convex, they may fall into local optima. For that reason, the objective function should be sufficiently smooth and the first or sometimes second derivatives should be present. Some classical examples of this strategy are Newton's method and hill climbing, which is also a root-finding algorithm. On the other hand, one of the modern examples is the conjugate gradient method. This strategy is widely used to solve unconstrained optimization problems such as energy minimization [22].

#### 2.2.2. Derivative-Free Algorithms

Unlike the previous one, this method only requires the value of the objective function, not the information of the derivative. If some discontinuity exists in cost functions, derivative-free algorithms may act in a more efficient manner. The Hooke-Jeeves pattern search method is one such method. It incorporates the past history of iterations in producing a new search direction [23]. Some other examples of this type of algorithms are the trust-region method and the Nelder-Mead downhill simplex method [24].

#### 2.2.3. Bio-Mimic Algorithms

Modern optimization algorithms are often nature-inspired/bio-mimetic, and they are suitable for global optimization. There exist a diverse range of bio-mimic or metaheuristic algorithms for optimization, including Particle Swarm Optimization (PSO) [25], Genetic Algorithm (GA) [26], Ant Colony Optimization (ACO) [27], Cuckoo Search (CS) [28], Bat Algorithm (BA) [29], *etc.* The right choice of an optimization algorithm can be crucially important in finding the right solutions for a given optimization scenario.

## 3. Rational for Optimization in WSNs

### 3.1. Wireless Sensor Networks and Optimization

**Figure 2.**
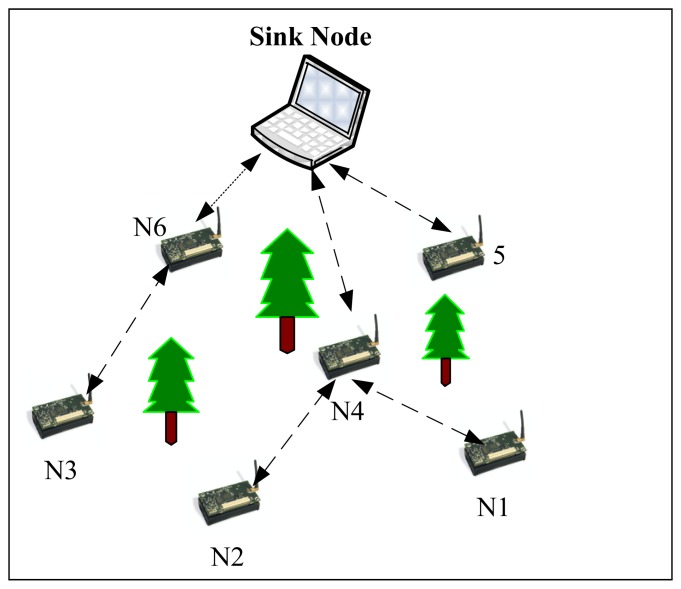
Architecture of a general wireless sensor network.

A WSN typically has little or no infrastructure. A sensor network is created with a large number of sensor nodes, which are deployed either inside the monitoring substance or very close to it (as shown in Figure 2) [30]. Unlike traditional networks, a wireless sensor network has its own design and resource constraints. Sensor nodes carry very limited, non-replenishable power sources. As a result, while traditional networks focus more on achieving high quality of service (QoS), sensor network protocols have to focus primarily on power conservation issues. Other resource constraints include low bandwidth, short communication range, and limited processing and storage in each node.

All the above mentioned issues are directly related to the optimization problem. Maximizing the lifetime, ensuring the QoS along with security is not an easy task. Furthermore, often these three issues contradict each other. If we want to ensure energy efficiency we have to compromise on QoS and security. If QoS is assured, then the other two issues may lack proper awareness.

So, from the optimization point of view of WSNs, the right choice of the optimizer or algorithm for WSN problems is very important. The algorithm chosen for an optimization task will largely depend on the nature of the algorithm, the type of the problem, the desired quality of solutions, the available resources, time constraints, *etc.* The nature of an optimizer may determine if it is appropriate for a particular type of problem. For instance, derivative-based algorithms such as hill-climbing are not appropriate for optimization problems whose objective is discontinuous. On the contrary, the type of problem we are trying to solve also can play role in determining which algorithm to choose. If the objective function of the problem is highly nonlinear and multimodal, the classical algorithms are not appropriate, as they are local search algorithms. Most WSNs suffer from huge resource constraints, and most of the problems that are to be optimized are NP-hard problems, so the cost of simulators or mathematical programming engines used for linear, nonlinear and quadratic programming make them unattractive. As the problem size increases, the computational complexity of conventional methods grows exponentially. This is the main inspiration for choosing bio-mimetic algorithms (global optimizers) such as PSO, GA, ACO, CS, *etc.*

### 3.2. Domains of Optimizations in Wireless Sensor Networks

As we mentioned earlier, in WSNs there are three key issues that are highly needed to be optimized, namely energy efficiency, QoS, and security. Again, these have some conflicting issues. For example, if we want to ensure timeliness (QoS), we need to compromise on the lifetime (energy efficiency) of the network. The same goes for security-related parameters. If we want to have transaction with highly secure data over a network, we need to compromise with either QoS or lifetime, or in some extreme cases with both of them, by adopting complex and energy consuming security solutions. Therefore a proper trade-off has to be made between these highly sensitive and conflicting areas of wireless sensor networks. An insignificant amount of research has been focused in this particular area which encompasses the overall optimization of these three issues simultaneously. There are obviously some high quality works focusing on each individual area and the progress and pace of research has been very fast, but a research loophole exists when it comes to the question of optimizing all three issues to make a better wireless sensor network in the real sense. Here we will discuss these issues and try to find out whether they can exist in symbiosis or not.

#### 3.2.1. Energy Efficiency *vs.* QoS

Wireless sensor networks are primarily characterized by their small amount and non-replenishable energy supply. Advancements in wireless sensor networks have led to a number of new protocols explicitly designed for sensor networks where energy awareness is the main consideration. Some of the research works have been done focusing on routing protocols since they might differ depending on the applications. Routing protocols aim to provide uniform energy dissipation during transmission to the sink node. This energy is mainly used for transmitting and receiving sensor readings, which are energy hungry operations. If all the sensors want to communicate with the BS directly, then it could result in the premature death of the whole network, so without a proper communication reduction strategy the whole system might be in jeopardy. Hence, the need for energy efficient infrastructure is becoming very important since it impacts the network's operational lifetime.

Almost all of the routing protocols can be classified as data-centric, hierarchical or location-based. Few of these protocols are aware of QoS. Along with the routing function, they include routing approaches that are based on general network-flow modeling and protocols that strive to meet some QoS requirements, but keeping in mind the resource constraints, the network QoS may suffer from lack of computing and communication resources [31]. As an example, if a number of nodes want to transmit *l* bit of message over the same WSN, they have to compete for the limited bandwidth that the network provides. As a result, some data transmissions may experience long delays, resulting in poor level of QoS, especially in real time applications. Also due to the limited memory size of the nodes, some data packets may be dropped or lost before they even reach the destination/sink.

Data redundancy is another important QoS parameter related to the issues of energy constraints. WSNs are characterized by sensor data high redundancy. However, while the redundancy in the data does help loosen the reliability or robustness (QoS) requirements of data delivery, it unnecessarily spends much energy. Data compression can be a good solution in providing energy efficiency by removing the data redundancy, but this energy efficiency can come at the cost of reduced reliability and increased delays and distortion.

Thus, in a sense QoS is also related to the issues of energy efficiency. In fact, energy efficiency itself is a QoS parameter. Somehow these two conflicting but incorporated areas need to be dealt with utmost intelligence and in this case biological intelligence can play a vital role. In order to achieve a prolonged network lifetime with a proper balance of power and suitable QoS support, energy loads must be evenly allotted among all the nodes. As a result, the energy of a single sensor or a small set of sensors will not be drained much earlier than others. QoS management must take this factor into account.

#### 3.2.2. QoS and Security

Security and QoS are two critical network issues in WSNs. Security mechanisms are used to maintain confidentiality, integrity, and availability of the services provided by WSNs. On the other hand, in real time applications QoS enables the sensed data to be delivered within a bounded delay period. QoS research has focused for several years on problems such as packet loss rate, throughput, bandwidth guarantees, jitter, delay, and other performance-related parameters when transmitting data over a specific network. But interestingly the issue of security is rarely mentioned. In fact, the earlier approaches were such that, if someone wants QoS and network performance for the data traffic, security cannot be part of the equation.

So the question is still out there, whether the network QoS and security are still orthogonal to each other or should one consider security as another QoS parameter and integrate it with the performance-related QoS parameters. So, the main question is, “Can QoS and security coexist or not”? Our conjecture is that network QoS and security can coexist if correct security policies are used in the right places.

The mechanisms of security and QoS are interdependent. Security mechanism choices impact the effectiveness of QoS and *vice versa*. QoS requires security mechanisms to ensure appropriate service assignment. A poor level of security measurement selection can massively jeopardize the performance of the network. Both services are necessary for safe and sound network operations. If we do not have information about QoS requirements, a poor choice of encryption endpoints may reduce the effectiveness of QoS performance. On the other hand, without information on security requirements, a poor assignment of QoS performance parameters may lead to denial of service for vital but low bandwidth data.So lack of good understanding of these interactions and inappropriate service level selection can leak extra information about the importance of packets in the traffic stream, but clever manipulation of QoS parameters like data freshness/timeliness might even help to reduce leakage of information through channels. Therefore, both services must be considered together when designing and implementing a network infrastructure to achieve the best possible security and QoS levels.

#### 3.2.3. Energy Efficiency *vs.* Security

As wireless sensor networks are rapidly growing, the need for effective security approaches are also becoming important. Many sensor networks have mission-critical tasks and may interact with sensitive data such as military applications, so it is clear that security needs to be taken into account at the time of design. While WSNs derive from wireless *ad hoc* networks, due to inherent resource and computing constraints, security in sensor networks poses different challenges than the traditional network security.

All security approaches require some amount of resources for implementation, including data memory, storage and energy to run the sensors. However, these resources are very limited in wireless sensor nodes and they are non-replenishable, so in order to build an effective security mechanism, it is necessary to limit the code size of the security algorithm.

For example, a common sensor which is relatively cheap and widely used in the research area is the TelosB. It has 16-bit, 8 MHz RISC CPU with only 10 KB RAM, 48 KB program memory, and 1,024 KB flash storage [32]. With this limitation, the operating system (OS) built for the sensors must also be quite small. The total code space of TinyOS, the standard OS for wireless sensors, is approximately 4 KB and the core scheduler occupies only 178 B, so, the code size for any security- related code must also be small.

Limited power or energy is the biggest constraint for wireless sensor networks. We assume that once the sensor nodes are deployed in a network, they cannot be easily replaced or recharged. Thus, charge taken with them to the final location must be conserved to extend the lifetime of the individual sensor node along with the sensor network. When implementing a cryptographic function or security protocol within a network, the energy impact of the added security code must be also taken into account. The extra power consumed by sensor nodes due to the addition of security, is related to the processing required for security functions like encryption, decryption, data signing, *etc.*, so a fine tuning of these two is essential.

### 3.3. Co-existence of Energy Efficiency, QoS, and Security

After all this discussion the question may arise on the coexistence of these three issues in a WSN application. Our conjecture is that it mostly depends on the application type. As an example Body Area Network (BAN) applications have significant legal, financial, privacy, safety, and real time implications. Hence, data freshness/timeliness, privacy, confidentiality, authentication, authorization, and integrity are their most fundamental requirements. Moreover, being a type of wireless sensor network, energy efficiency in most applications of BAN is a prerequisite, so the coexistence of energy efficiency, QoS (timeliness), and security is necessary, but as we discussed earlier in most cases these are conflicting issues which require a trade-off between them and optimization can be a useful tool in making these trade-offs.

**Figure 3.**
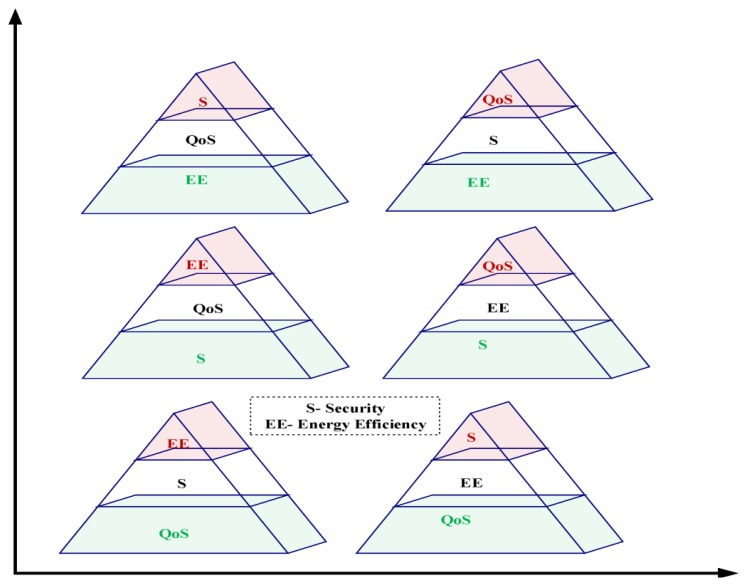
A pyramid view of how optimizations of Energy Efficiency, QoS and Security in a wireless sensor network are related to each other.

From the pyramid view of Figure 3, we can easily notice the self contradicting nature of the three areas of a wireless sensor network in three layers. In the lowest two pyramid view, it is clear that if we want to ensure high amount of QoS then eventually the other two parameters will be affected. In the middle pyramids we can see the same scenario with security as the most important issue. Like the previous one, here QoS and energy needs to be compromised. In the upper two pyramids energy efficiency is the most prioritized issue, so QoS and security parameters are highly compromised.

## 4. Survey of Existing Works

WSNs pose unique characteristics such as extremely resource-constrained, large scale deployment, etc. To solve the issues of WSNs with bio-mimetic approaches, researchers have proposed several algorithms over the last two decades. In the following subsections we will try to elaborate and give an insight into some of the leading methods, namely PSO, GA, ACO, *etc.*, which are widely used in the WSN arena.

### 4.1. PSO in WSNs

Particle Swarm Optimization (PSO) was invented by Kennedy and Eberhart in 1995 [25]. They were trying to simulate the amazing controlled motion of a swarm of birds flying in one direction. In PSO, particles regulate their information (flying directions) with its own flying experience as well as their neighbors' flying experience. In a word it combines self-experience with social experience [33], so the basic PSO was a social behavior simulator. It consists of a swarm of *s* candidate solutions called particles. Several revised versions of PSO have emerged with a range of concepts and applications including WSNs. A number of parameters such as inertia weight (*w*) and confidence factors (*c*_1_, *c*_2_) were added later on [34,35] to improve the efficiency of the method. After several improvement processes it was understood that the technique can be used as a population-based optimizer and it can solve stochastic nonlinear optimization problems in a cheaper way. A more recent study on variations and taxonomy of PSO is presented in [36].

**Figure 4.**
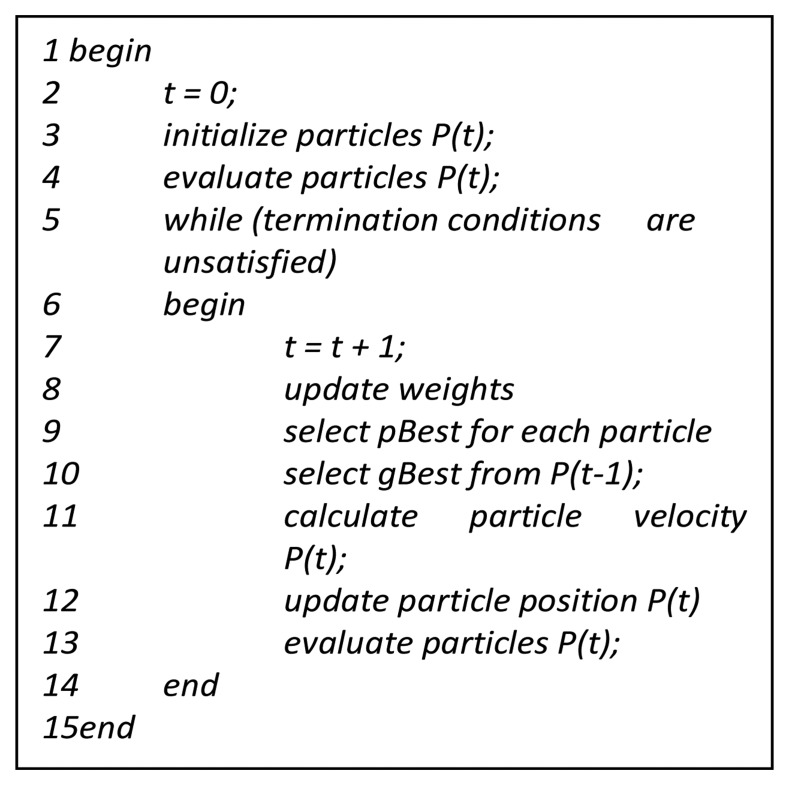
Pseudo code of PSO.

Generating particles' position and velocities, velocity update, and position update- these three main steps defines the PSO algorithm. Here particle refers to a point in a D-dimensional search space that updates its position from one point to another based on related velocity updates. The *i*-th individual (particle) of the population, which is called swarm, can be represented in a D-dimensional vector as, *X_i_* =(*x_i_*_1_, *x_i_*_2_,…, *x_iD_*). The velocity or the position change for particle *i* is represented as *V_i_* =(*v_i_*_1_, *v_i_*_2_,…, *v_iD_*) and the best previously visited position of this particle is denoted as *P_i_* =(*p_i_*_1_, *p_i_*_2_,…, *p_iD_*). Symbol *g* represents the best particle in the swarm and *w* is the inertia weight. The particles are then manipulated according to the following two equations [37]:

(4) $$V_{id}^{n+1}=wV_{id}^{n}+c_{1}r_{1}^{n}\left(P_{id}^{n}-X_{id}^{n}\right)+c_{2}r_{2}^{n}\left(P_{gd}^{n}-X_{id}^{n}\right)$$

(5) $$X_{id}^{n+1}=X_{id}^{n}+V_{id}^{n+1}$$

where *d* = 1, 2, …, D, *i* = 1, 2, …, N and N is the size of the swarm and *n* = 1, 2, … denotes the iteration number. Two random numbers r1, r2 which are uniformly distributed in [0, 1] ensure good coverage. They also ensure the avoidance of falling into local optima which was a problem of the classical approaches. The inertia weight *w* manipulates the trade-off between exploration and exploitation abilities of the flying points. Another two important parameters are *c*1 (self-confidence factor) and *c*2 (swarm confidence factor). The stopping criterion of the algorithm depends solely on which type of problem it's going to deal with. One of the problems of PSO is the tendency towards a fast and premature convergence in mid-optimum points. A lot of effort has been made so far to solve this problem. A general pseudo code of PSO is shown in Figure 4.

Because of its inexpensive resource requirements, less occupation of memory and ability to solve stochastic optimization problems in a faster way, PSO is widely used in many types of WSN optimizations like energy aware clustering, optimal WSN deployment, node localization, data aggregation, *etc.*

#### 4.1.1. PSO in Design and Deployment of WSNs

The ubiquitous nature of wireless sensor networks is useful in performing measurements in harsh and inaccessible environments in an efficient way. Bio-mimetic techniques can be very handy in the designing and planning the deployment of nodes in such environments. The WSN design and deployment problem refers to the optimum positioning of the nodes and base stations (sink nodes) in a way that the coverage and connectivity with adequate energy efficiency is achieved [38]. In some cases, the sensor nodes that need to be placed are determined beforehand, like in health monitoring applications, whereas in disaster monitoring, such positioning is impractical and they are deployed in an *ad hoc* manner. Sensors deployed in an optimal manner can guarantee adequate QoS, prolonged lifetime, and secure communication [39].

*Node Positioning* in WSN is of two types, namely stationary and mobile node positioning. In [40] the authors tried to minimize the area of coverage holes via a centralized PSO-Voronoi algorithm for stationary node positioning. In this paper the coverage problem caused by limited sensing range (limited number of sensors) has been tackled using PSO and Voronoi diagrams. The method is based on the principle that if a sensor covers every point of the region-of-interest (ROI) then the whole ROI is covered. The Voronoi diagram is used to assess the fitness of the WSN's coverage. Based on this fitness, a PSO searches the most optimal position of the sensors. This PSO scheme finds close to optimal coverage, but ignores the complexity of determining Voronoi polygons [10].

Another work on stationary node positioning is presented by the Hu *et al.* in [41]. They proposed a topological planning method named PSO-Traffic (a binary PSO) for real world traffic surveillance (a main subsystem of intelligent transportation systems (ITS)) and the sensors are plotted around the 2nd Ring Road in Beijing. The concept of small world is used in the study [42]. They used a large number of camera-loaded sensor nodes which are situated by the roadside. The PSO method is used to calculate the global best distribution of the nodes with the large radius. The target was to find optimal allocation of high power transmitters to existing nodes so that maximum coverage is achieved with minimized cost. This technique has ensured the symmetric distribution of high power transmitters, minimization of system cost and improvement in network performance.

In [43] a sequential form of PSO is elaborated for a maritime surveillance application. The goal is to find out the optimal placement of sonar sensors so that detection coverage is maximized in a fixed volume *V* which represents a maritime region. The article states that the method can achieve about 6% better coverage compared to the standard PSO.

Apart from the stationary node positioning in [44], Li *et al.* have proposed a hybrid approach for positioning stationary and mobile nodes to address the problem of coverage in WSNs. A modified PSO named particle swarm genetic optimization (PSGO) is used here. PSGO imports selection and mutation operators in PSO to overcome the premature fault of classical PSO. After the initial random deployment of nodes, the authors proposed to redeploy the mobile robots according to the node density for repairing the sensing coverage hole. It is shown by the simulation that the WSN employing the mobile robots can improve the QoS in sensing coverage by as much as 6% over the stationary WSN, but it necessitates mechanisms for obstacle avoidance and location awareness.

Another approach is presented in [45]. This paper proposes a dynamic deployment algorithm which is named “virtual force directed co-evolutionary particle swarm optimization” (VFCPSO). This algorithm combines the co-evolutionary particle swarm optimization (CPSO) [46] with the VF algorithm. In virtual force (VF)-based dynamic deployment, the sensors iteratively move based on virtual attractive forces or repulsive forces from other nodes. The new position of a sensor is computed in such a way that it moves in the direction of VF by step size proportional to its magnitude. Authors report that the proposed VFCPSO is competent for dynamic deployment in WSNs and has better performance with respect to computation time and effectiveness than the VF, PSO and VFPSO algorithms.

**Figure 5.**
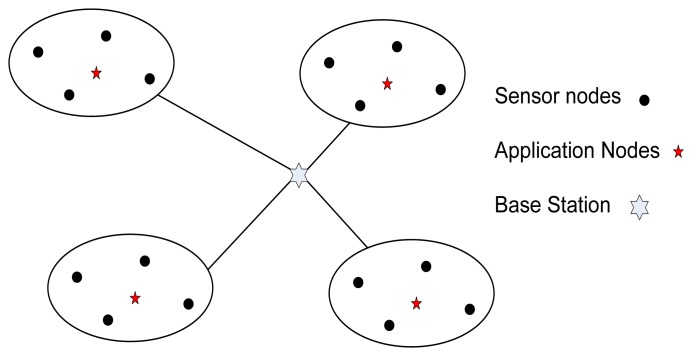
A two tier architecture of WSN.

*Base Station Positioning* is another important factor for designing WSNs. The base station is usually assumed to have unlimited energy and powerful processing capability. In [47], a two-tiered wireless sensor network has been considered (see Figure 5) and an algorithm based on particle swarm optimization (PSO) has been proposed for finding the multiple base stations. The two tier network consists of nodes that can communicate only with the application nodes they are assigned to. Application nodes possess long-range transmitters, high-speed processors, and abundant energy. This method aims at determining positions of base stations so that the total of distances of application nodes to their nearest base stations is minimum. This deployment requires minimum transmission power and assures maximum network life.

The proposed algorithm first randomly generates an initial group of particles, with each particle representing a possible multiple base-station location solution. Each particle also allocates a velocity for changing its state. System lifetime is used as the fitness function to evaluate each particle. Both the local optimal value pBest and the global optimal value gBest are then used to guide the search direction. When the termination conditions are achieved, the final gBest will be output as the location of the multiple base stations. Experiments have also been made to show the performance of the proposed PSO approach and the effects of the parameters on the results. In summary, the proposed algorithm can help to find good BS locations to reduce power consumption and maximize network lifetime in two-tiered wireless sensor networks.

The authors in [48] presented another application of PSO. The target is to achieve the optimal path for sink node (base station) movement across the sensor field. The research defines node throughput as the average number of data units forwarded by the sensor node in a time slot. The overall throughput of the sensor field is the aggregated throughput at a given sink node location. On the other hand, the average throughput is the average of the aggregated throughput collected from each point. From the simulation it is seen that average throughput degrades notably when the sink is moving with a large number of sensor nodes but achieves significant network coverage. Authors in [48] showed that, when the number of sensors are 3, the average throughput is 0.12099 and the field radius coverage is 0.015 m, but when the number of sensors are 100, the average throughput degrades but the coverage is increased to 0.500 m.

Another work on base station positioning was demonstrated in [49]. Here they focused on one of the major issues of WSN that is the trade-off between the total Capital Expenditure (CAPEX) to implement the network and quality of service (QoS). The higher the number of base stations (BSs), the higher are the chances of availability of the network for the user. In this paper, authors propose to adapt the PSO algorithm in a non-conventional way to solve the maximum coverage problem. The algorithm determines the placement of the BS taking into account the demand distribution in order to maximize the QoS of the WSN.

In [14] and [50] Latiff *et al.* proposed two energy-efficient protocols for the movement of mobile base stations in WSNs using PSO. In [14] an application specific scenario (environment monitoring) is considered. As a result of introducing mobile BS, the energy efficiency, lifetime and data delivery of WSNs is greatly improved. Simulation results showed that the protocol can improve the network lifetime, data delivery and energy consumption compared to existing protocols. Another energy-efficient protocol for the repositioning of mobile base stations using PSO in WSNs named PSO-BSP is presented in [50]. This work is concerned with repositioning the BS in a network with clustered sensor nodes. The repositioning of the BS can be precious in spreading the traffic by increasing hops and the feasibility for achieving the timeliness requirements. Results indicated that the proposed protocol showed gains in energy efficiency compared to protocol [11], which did not consider the BS repositioning.

#### 4.1.2. PSO in Node Localization

Creating location awareness in deployed nodes in WSNs is known as node localization [51]. An obvious but unattractive method of localization is to equip each node with a global positioning system (GPS). Many WSN localization algorithms approximate locations of sensors using a previous knowledge of the coordinates of special nodes called beacons. WSN localization is a two phase process: ranging phase and estimation phase. In first phase all the normal nodes estimate their distances from beacons, the special nodes, using signal propagation time or strength of the received signal [52]. Precise measurement of these parameters is not possible due to noise so the result of such localization is inaccurate. In the estimation phase, position of the target nodes is estimated using the ranging information either by solving equations, or by an optimizer like PSO, which minimizes localization error [10]. Node localization is a multidimensional optimization problem and it can be handled with bio-mimic methods like PSO.

In [53] Gopakumar *et al.* have proposed a PSO based localization scheme. The objective is to estimate *x* and *y* coordinates of *n* nodes in a network of *m* nodes deployed in two dimensional plane. The remaining (*m* – *n*) nodes are anchor nodes. Hence for a 2-D localization problem, a total of 2*n* unidentified coordinates, *θ* = [ *θ_x_*, *θ_y_*]; where *θ_x_* = [*x*_1_, *x*_2_, …, *x_n_*] and *θ_y_* = [*y*_1_, *y*_2_, …, *y_n_*] are to be estimated using anchor node coordinates [*x_n_*_+1_, …, *x_n_*_+_*_m_*] and [*y_n_*_+1_, …, *y_n_*_+_*_m_*]. If (*x*, *y*) are the coordinates of the target node to be determined, then the distance between the target node and the *i*^th^ anchor node *d_i_* will be:

(6) $$d_{i}=\sqrt{{\left(x-x_{i}\right)}^{2}+{\left(y-y_{i}\right)}^{2}}$$

The BS runs PSO to minimize the objective function which is defined as:

(7) $$f\left(x,y\right)=\frac{1}{M}\sum_{i=1}^{M}{\left(\sqrt{{\left(x-x_{i}\right)}^{2}+{\left(y-y_{i}\right)}^{2}}-{\hat{d}}_{i}\right)}^{2}$$

where (*x*, *y*) is the node location that needs to be determined and (*x*_1_, *y*_1_) are the coordinates of the *i*^th^ anchor node. M ≥ 3 is the number of beacons or anchor nodes within transmission range of the target node. Here *d̂_i_* is the measured value of *d_i_* between the beacon *i* and a node (calculated under noise conditions). The variance of noise affects the localization precision. The method works well if the beacons have sufficient range or the beacons are plenty in number. Simulation showed that the localization error is more than halved with respect to simulated annealing [54] in all experiments, but it needs to be mentioned that in this method BS requires distance estimation from all nodes to all beacons. This results in congestion and massive expenditure of energy in WSNs.

An addition to the above work is that reported in [55] by Kulkarni *et al.*, which uses a bacterial foraging algorithm along with PSO. It is focused on range-based distributed iterative node localization. In this scenario the target node that has three or more beacons in its hearing range runs PSO to minimize the localization error and estimates its own x and y coordinates in a plane mission space. The localized nodes act as beacons themselves in the next iteration. This continues iteratively till all the nodes get localized. This method can localize all nodes that have three localized nodes or beacons in their range. This approach can lessen inaccuracies due to flip ambiguity based on some conditions. The work compares PSO with bacterial foraging algorithm with normal PSO. It is reported to show more efficiency in terms of searching capability. On the other hand, the bacterial foraging algorithm is reported to be less memory intensive and more accurate.

In [56] Low *et al.* have proposed a PSO-based distributed localization scheme that does not involve beacons. In this paper, a comparatively inexpensive localization scheme is presented. It is based on the measurements from a pedometer and communication ranging between neighbouring nodes. For ease of testing, a person equipped with a pedometer and an electronic compass is considered as the deployment agent. The pedometer provides the distance and the electronic compass gives the angle of heading with respect to the magnetic north. The proposed system works well in a sparse network. The localization information is obtained through a probability based algorithm that requires the solving of a nonlinear optimization problem. To obtain the optimum location of the sensor nodes, the PSO scheme that can be realized with a microcontroller for real time application is investigated in this paper. Experimental results show that the localization results of PSO and GNA are only slightly different. For the similar given measurements, both schemes are able to find similar maxima. The slight variations are due to the different stopping criteria. From the simulations, the run time is also found to be comparable. Nevertheless, it is to be noted that the PSO is more robust as it constantly yields a distinctive result whilst the GNA involves matrix inversion during its iteration.

In another work, Low *et al.* have proposed a localization scheme for unknown emitter nodes in a WSN [57]. This system assumes that there are four beacon nodes with known locations. One or more unknown nodes transmit RF signals that can be received by the anchor nodes. A node at location *O* in the sensor field can estimate its distance from a beacon as where *P* is the power transmitted by the beacon and *P*_0_ is the power at unit distance *d*_0_. The only available information to the system is the received signal strength indicator which is in general not very accurate. To obtain better estimated sensor node locations, the PSO scheme that can be realized in real time is investigated in this paper. It is observed from the experimental results that the calculated loss exponent *α* (a particle member) is between 3 to 5, which is a reasonable value as compared to other published research works. In general, the experimental errors are reasonable and are consistent with the simulation results. The results also validate that whenever the emitter node is near the centre of the rectangle, the error tends to be smaller. However, if the emitter node is moved closer to the area of the rectangle, the error increases significantly.

#### 4.1.3. PSO in Energy Aware Clustering

WSNs are mainly characterized by their limited energy supply. Hence, the need for energy efficient infrastructure is becoming increasingly more important since it impacts the network operational lifetime, so balanced usage of energy is a critical issue in WSNs. Typically communication is the most energy-expensive act that nodes perform. Energy required to transmit *l* bits of data varies exponentially with transmission distance *d*, so it is common to use multi-hop communication. Routing in WSN refers to the selection of a definite path for a packet to go from a source node to a sink. The hierarchical routing has its all nodes clustered into groups. A cluster-head (CH) acts as the main node in a particular cluster that collects all the data from other non cluster head nodes. A node that acts as a CH for a long duration loses its energy prematurely, so an optimal cluster-head election mechanism is essential. Again, proper CH assignment influences network performance and longevity. Heinzelman *et al.* proposed low energy aware clustering hierarchy (LEACH) which is a simple and efficient algorithm [58]. As we already know from previous discussion that clustering is an NP-hard problem, which bio mimetic optimization methods like PSO can handle efficiently.

The first PSO approaches in selecting CH efficiently can be found in [10]. Tillet *et al.* proposed a method using PSO that tries to equalize the number of nodes and candidate CH in every cluster of a network, with the target of minimizing the energy spent by the nodes while maximizing the data transmission. However, no comparison with other benchmark clustering strategies has been addressed.

In [11] the authors consider both available energy in nodes and physical distances between them and their CHs. They defined a new cost function, with the purpose of minimizing the intra-cluster distance and optimizing the energy consumption of the network at the same time. Proposed protocol selects a high-energy node as a CH and produces clusters that are equally placed throughout the entire WSN field. The performance of the protocol is later compared with the well known cluster-based protocols like LEACH and LEACH-C (an improved version of LEACH) and simulation results demonstrated better network lifetime and data delivery at the BS. The fitness function for the centralized PSO (PSO-C) is defined as *cost* = *β* × *f*_1_ + (1 − *β*) × *f*_2_, where *f*_1_ is the maximum average Euclidean distance of nodes to their associated cluster heads and *f*_2_ is the ratio of total initial energy of all nodes to the total energy of the cluster-head candidates in current round. The key difference between the works [10] and [11] is the application of PSO to choose the optimal nodes as cluster heads to extend the network lifetime.

In [59] authors Chunlin *et al.* proposed a revised PSO to one clustering algorithm named Weighted Clustering Algorithm (WCA), in sensor networks. WCA is a recent clustering algorithm, which was revised to be suitable for dense mobile nodes distribution here. Then, Divided Range Particle Swarm Optimization (DRFSO) algorithm was applied to this revised WCA optimization. The particles were divided into groups running in four neighbourhood nodes simultaneously. The approach restricts the number of nodes to be catered by a CH to ensure efficient medium access control (MAC) functioning. It has also the flexibility of assigning different weights and takes into account a combined effect of the ideal degree, transmission power, mobility, and battery power of the nodes. Simulation study showed competent and effective results over other methods, especially when the distribution of mobile nodes is dense.

Four variants of PSO were proposed by Guru et al. in [60] for energy aware clustering, namely PSO with time varying inertia weight, PSO with time varying acceleration constants, hierarchical PSO with time varying acceleration constants, and PSO with supervisor student mode. In variant, the inertia weight w is decreased linearly from 0.9 in first iteration to 0.4 in the last iteration. In PSO with time varying acceleration constants, inertia weight is set constant, and acceleration constants *c*1 and *c*2 are varied linearly in every iteration, so the particles move in large steps initially but the step size reduces in every iteration. In hierarchical PSO with time varying acceleration constants method, the particle update is not influenced by the velocity in previous iteration. Thus, re-initialization of velocity is done when the velocity stagnates in the search space. Therefore, a new set of particles is automatically generated according to the behaviour of the particles in the search space, until the convergence criterion is met. Lastly, the PSO with supervisor student variant updates its position according to Equation (8). This method introduces a novel parameter called momentum factor (mc) which updates the positions of particles (refer to Table 1 for other notations). In this strategy the velocity of the particle is updated only if its fitness at the present iteration is not better than that of previous iteration. The velocity acts as a pilot (supervisor) by getting the accurate direction, whereas the position (student) obtains a right step size along the direction. A detailed comparative analysis of the algorithms for optimal clustering is presented. This scheme considers only the physical distances between nodes and their assigned cluster-heads, but not the energy available to the nodes:

(8) $$x_{id}^{n+1}=\left(1-mc\right)\times x_{id}^{n}+mc\times v_{id}^{n+1}$$

Cao *et al.* [61] have considered a slightly different case in which a node and its CH are engaged in a multi-hop communication. The proposed algorithm synthesized the intuitionist advantages of graph theory [62] and optimal search capability of PSO [63]. They calculated the distance based on minimum spanning tree of the weighted graph of the WSN. The CHs were elected by maximum residual energy and in turns and by probabilities separately. The best route between a node and its CH is derived from all the optimal trees on the basis of energy consumption. The authors concluded that the network lifetime has almost nothing to do with the BS location or the residual energy of the node. Once the topology of the network is decided, the lifetime is settled. They also mentioned that there are two ways to improve the network lifetime. One way is to reduce the energy consumption for transmitter or receiver start up. Other way is to optimize the network topology. The performance was compared with three mechanisms of CH election: energy-based, auto-rotation-based, and probability-based. The results show that the PSO-based clustering methods ensure prolonged network lifetimes.

#### 4.1.4. PSO in Data Aggregation

WSNs consist of sensor nodes with sensing and communication capabilities. When a WSN is used to monitor a region, each sensor node in the network collects local observations and sends compressed or partially processed data (a summary) to the fusion centre. The fusion centre (data aggregation center) uses the summary and applies specific decision fusion rule to make the final decision. The main goal of data aggregation is to gather and aggregate data in an energy efficient way so that network lifetime is improved [64]. Data fusion is a distributed and repetitive process which is quite suitable for PSO. Effective data aggregation influences network performance. Therefore, it is reasonable to choose PSO to control the parameters of fusion. PSO has provided optimization in several aspects of data aggregation as follows.

In [65], authors address the problem of optimal power allocation through a constrained PSO. Their algorithm uses PSO to determine optimal-power allocation in the cases of both independent and correlated observations in a Gaussian sensor network. The optimal power scheduling scheme indicates that the sensors with poor observation quality and bad channels should be inactive to save the total power expenditure of the system. The wireless link between sensors and the fusion centre is assumed to undergo fading. The coefficients are assumed to be available at the transmitting sensors. The objective is to minimize the energy expenditure while keeping the fusion-error probability under a required threshold. The authors presented that the probability of fusion error performance based on the optimal power allocation scheme determined by PSO outperforms the uniform power allocation scheme, especially in case of a large number of nodes or when the local observation quality is good.

Veeramachaneni *et al.* presented a hybrid approach of ant-based control and PSO for hierarchy and threshold management for decentralized serial sensor networks in [66]. The performance of the decentralized sensor network is sensitive to the design of thresholds for individual sensors and to the communication hierarchy among sensors. The PSO is used to determine the optimal thresholds and decision rules (fusion rules) for the sensors while the ant colony optimization algorithm determines the hierarchy of sensor decision communication. The results achieved are compared to the fixed hierarchy and a traditional approach using the best performing sensor at the top of the hierarchy. Probabilistic measures including probability of error and Bayesian risk are adopted to evaluate the performance of the sensor network. Results show 40% performance improvements in terms of Bayesian risk value.

In [67], Veeramachaneni *et al.* present a binary multi-objective PSO for optimal sensor management of multiple sensor networks. PSO is modified to optimize two objectives: accuracy and time. PSO searches the configuration space and finds an optimal configuration. An additional objective of time has been added to increase the complexity. The particle swarm algorithm is modified to solve this multi objective problem for a few different priorities of the objectives. Bayesian decision fusion framework as in [68] is used to fuse the decisions from multiple sensors. The output of the algorithm is the choice of sensors, individual sensor threshold, and the optimal decision fusion rule. Results show the capability of the algorithm in selecting optimal configuration for a given requirement consisting of multiple objectives. This algorithm can be used for managing a network of radars, which detect the presence of an aircraft, rain clouds, missiles, *etc.*

The authors in [69] presented a multi-source temporal data aggregation model in WSNs, including feature selection and data prediction. Data aggregation has emerged as a basic approach in wireless sensor networks (WSNs) in order to reduce the number of sensor node transmissions. This work proposes an energy-efficient multi-source temporal data aggregation model called MSTDA. This model is deployed at both the base station (BS) and the node. MSTDA helps to find out potential laws according to historical data sets. In this model, a data prediction algorithm based on improved BP neural network with PSO (PSO-BPNN) is proposed. Feature selection based on PSO extracts the essential data from thousands of sample data, the simplified datasets are then employed by PSO-BPNN for prediction. The experiments on the dataset which comes from the actual data collected from 54 sensors deployed in the Intel Berkeley Research lab showed good results.

Jiang *et al.* in [70] designed a linear decision fusion rule and proposed a way of controlling the parameters of the model taking the advantage of the constrained PSO. It is obvious that the decision-making capability of each node is different due to the different signal noise ratios and some other factors, so a specific sensor's contribution to the global decision should be constrained by this sensor's decision-making capability. Based on this idea, a novel linear decision fusion model for WSNs was established. In the model, the integrated contribution of local decisions is computed with a linear equation which is made up with local decision weights and local decisions. Then the integrated contribution is compared with a threshold in the fusion centre. Later on, according to the comparison results, the final decision is made. In order to get the smallest error probability, constrained PSO is employed to find out the optimal local decision weight and the threshold. The simulation results indicated that the linear decision rule and the parameter optimization method are efficient to get very high accuracy.

Available PSO solutions to the problems discussed so far are summarized in Table 2.

### 4.2. Ant Colony Optimization

Like some other swarm intelligence approaches that take inspiration from the social behaviors of insects and animals, ants have inspired a number of methods and techniques among which the most widely studied is the general purpose optimization technique known as ant colony optimization (ACO). ACO is a method which is inspired from the foraging behavior of some ant species. These ants deposit pheromonee on the ground in order to mark their paths from the nest to food that should be followed by other members of the colony. This algorithm has a mechanism for solving discrete optimization problems in various engineering domains.

Initially the optimization algorithm was proposed by Dorigo in 1999 [71]. The primary idea has since been widely researched and diversified to solve a broader class of numerical problems. The ACO heuristic algorithm was later introduced by Dorigo and his collaborators for solving some combinatorial optimization problems [72], such as the traveling salesman problem (TSP) [73]. The general foraging behavior of ants is described below [27]:

1. The first ant finds the food source, via any way, and then returns to the nest, leaving behind a pheromone trail.
2. Ants indiscriminately follow possible ways, but the strengthening of the runway makes it more attractive as the shortest route.
3. Ants take the shortest route; long portions of other ways lose their trail pheromones.

**Figure 6.**
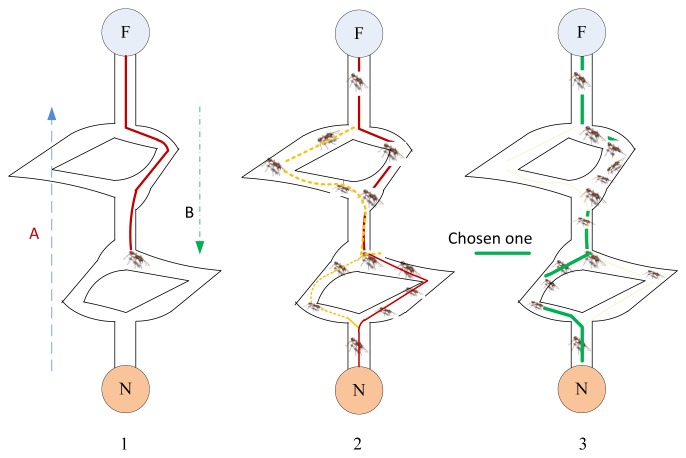
Ants follow the minimal path from nest to food source.

For example if there two paths A and B exist between a nest and a food source (see Figure 6), and *n_A_*(*t*) and *n_B_*(*t*) are the number of ants use them at time step *t*, respectively, then the probability of ant choosing path A (*P_A_*), at the time step *t* + 1 is given by the following equation:

(9) $$P_{A}\left(t+1\right)=\frac{{\left(c+n_{A}(t)\right)}^{\alpha }}{{\left(c+n_{A}(t)\right)}^{\alpha }+{\left(c+n_{B}(t)\right)}^{\alpha }}=1-P_{B}(t+1)$$

where *c* is the degree of attraction of an unexplored branch, *P_B_* is the probability of choosing path B, and α is the bias to using pheromone deposits in the decision process (*α* ≥ 0). An ant chooses between the path A or path B using the decision rule: if *U*(0, 1) ≤ *P_A_*(*t* + 1) then choose path A otherwise choose path B. U is a random number having uniform distribution in the range (0, 1).

Researchers have shown that ACO performs well in solving stochastic time-varying problems (e.g., routing in networks) because of its flexibility and decentralized nature. ACO presented many desirable features in solving dynamic and distributed routing problem because of their similarities between ants' foraging and routing [8]. The following section reviews the recent research and implementation of ACO wireless sensor network field.

#### 4.2.1. ACO Based Routing Algorithms

Bio-mimetic methods like ACO are popular tools used by researchers to address the issue of energy-aware routing. Planning of energy-efficient protocols is vital for WSNs because of the constraints on sensor nodes' energy. Therefore, the routing protocol should be able to achieve uniform power dissipation during transmission to the sink node. In [75], an Energy Efficient Ant-Based Routing (EEABR) is designed to extend the life time of WSNs by decreasing communication overhead in the discovery phase. This is attained by way of two factors: energy and hop count. In addition, they use a fixed ant size to construct energy efficiency routes. Ants are generated proactively in EEABR at regular intervals and unicasted to the next hop SNs that is selected by a probabilistic rule. The protocol was studied by simulation for several WSN scenarios and the results clearly show that it minimizes communication load and maximizes energy savings.

Almshreqi *et al.* presented a self-optimization scheme for WSN in [76] which is able to utilize and optimize the sensor nodes' resources, to achieve balanced energy consumption across all sensors. Inspired by the colony of ants, they presented SensorAnt to use a new routing scheme to optimize the battery power of sensors participating in the paths to forward the data across the sensor networks. The objective function depends on multi-criteria metrics such as the minimum residual energy or battery power, hop numbers, and average energy of both route and the network. This method also distributes the traffic load of sensor nodes throughout the WSN leading to reduced energy usage, extended network life time, and reduced packet loss. Simulation results show that their scheme performs much better than Energy Efficient Ant-Based Routing (EEABR) in terms of energy consumption and efficiency. Other QoS metrics such as throughput, delay and packet loss are not addressed in this method.

For constructing optimal data-gathering routing structure in WSN and to improve the reliability of the tree structure in order to reduce the loss of efficient information, it is important to minimize the total energy cost of data transfer from the data-collecting region to a fixed sink for prolonging the lifetime of a WSN. To achieve the above two important objectives a Predication mode-based Routing Algorithm based on ACO (PRACO) to achieve energy-aware data-gathering routing is presented in [77]. Via load balancing in heuristic factors and acnovel pheromone updating rule in artificial ants, it can confer to the artificial ants the ability to adaptively detect the energy status of WSN and intelligently build the routing structure. Results show that the proposed method can effectively reinforce the robustness and effectiveness of routing structure by mining the temporal associability. Here ACO balances the total energy cost for data transmission. This contribution can improve the robustness of routing mechanism in WSN with the tradeoff between energy-saving effect and reliable structure.

A novel multipath routing protocol (MRP) based on dynamic clustering and ACO is presented in [78] for monitoring burst events in a reactive WSN. The authors introduced an objective function to carry out dynamic clustering. MRP improves the efficiency of data aggregation, thus, reducing the energy consumption. The improved ACO algorithm is used to search the optimal and suboptimal paths based on several metrics (e.g., path length and energy consumption of communication) that can balance the energy consumption among nodes. Moreover, a load balancing function is presented for dynamic selection of a path to transmit data. Test results showed that MRP achieved better load balancing and lower energy consumption, and overall maximizes the network lifetime.

The authors in [79] introduce routing algorithms implemented using two kinds of ACO and an improved ant system algorithm. A performance comparison of the three algorithms is carried out, mainly on the average energy consumption and the average delay. The simulation results show that the routing algorithm implemented by ACO can reduce effectively energy consumption. ACO proves to be an effective way to reduce the energy consumption and maximize the lifetime in WSNs.

In [80] a routing protocol defined as Biological inspired self-organized Secure Autonomous Routing Protocol (BIOSARP) is proposed to enhance the limitations of Secure Real-Time Load Distribution (SRTLD). SRTLD uses broadcast packets to perform neighbor discovery for every packet transfer every hop, and thus consumes high energy. The BIOSARP routing protocol depends on the optimal forwarding decision obtained by ACO. The pheromone value in ACO is computed based on end-to-end delay, residual energy, and packet reception rate metrics similar to SRTLD. The proposed BIOSARP has been designed to reduce overhead broadcast packet in order to minimize the delay, packet loss, and power consumption in WSN. In simulation study BIOSARP normalized overhead is 12.1% less as compared to E&D ANTS and achieves 14% higher delivery ratio with 9% less power consumption when compared to SRTLD.

The demand for real-time application in WSN is increasing day by day. So the quality of service (QoS)-based communication protocols are becoming a hot research area, specially in the case of wireless multimedia sensor networks (WMSNs). In WMSNs the transmission of imaging and video information requires both energy efficiency and QoS assurance (e.g., bandwidth, packet loss and delay constraints). In order to achieve the balance between energy-efficiency and QoS improvements Song *et al.* [81] presented a multiple QoS metrics hierarchical routing protocol (2ASenNet), with a combination of an improved ACO and artificial fish swarm optimization (AFSO) [82]. It adopted hybrid ant behavior to produce diverse original paths, while adding AFSO for the iterative process of the improved ACO and the optimization path was explored according to multiple QoS constrained. Experimental results indicated the efficiency of this novel approach, while simultaneously reducing the consumption of constrained resources as much as possible.

#### 4.2.2. ACO in WSN Deployment

Most of the previous works assume that in WSNs a sensing field is an open space. In [83] Wang *et al.* considered the sensing field as an arbitrary-shaped region with possible obstacles. They eliminated the constraints of existing results by assuming a random relationship between the communication range and the sensing range. In the Forbidden City of China, Li *et al.* [84] demonstrated a real application of a WSN system for relic protection. The authors developed a hardware named the EasiNet and the corresponding mesh-architecture of the system was constructed. A sensor deployment optimization tool based on ant colony optimization technology (DT-ACO) is proposed which guarantees the network connectivity, full optimized WSN sensing coverage, as well as minimized number of sensor nodes. A novel power-aware cross-layer scheme (PACS) is designed towards the challenge of adjustable lifetime and surveillance accuracy. It enables satisfactory system lifetime and surveillance accuracy in general applications. PACS was implemented into both sensing nodes and the sink. The sensing nodes use PACS to measure the degree of over-consumption, save the transmissions by data prediction, and adaptively adjust prediction accuracy. The sinks use PACS to cooperate with the sensing nodes when the prediction algorithm proceeds. The mesh architecture of the system achieves prolonged lifetime and an improvement on the data delivery rate than traditional methods [85,86] during real applications.

Li *et al.* have another relatively recent work [86] published in 2010, where they formulated the minimum-cost CGP *k* coverage problem in real sensor network system. An improved ant colony algorithm EasiDesign is proposed to achieve the approximate solution to this optimization problem. They mainly focus on two kinds of practical problems: optimizing the routing hops and avoiding obstacles. They first gave a new pheromone updating rule which considers not only the number of sensors but also the routing cost in the constructed solution, and then they designed an obstacle detection component to guide the ants to go around the obstacles. The obstacle avoidance and the routing cost trade off strategies ensure that the EasiDesign can work efficiently. The simulation results show that EasiDesign uses less sensor nodes than the existing works in the same scenario. The optimum configuration of key parameters in EasiDesign proves that it achieves better performance than the traditional ant colony algorithm. With routing optimization method, EasiDesign largely reduces system routing cost by a small number of redundant sensors. Like previous research work they have demonstrated the performance through a real sensor network system for the environment monitoring in the Forbidden City.

In [87] the authors considered the problem of sensor deployment to achieve complete coverage of the service region and maximize the lifetime of WSNs. They modeled the deployment problem as the multiple knapsack problem. The ACO algorithm provides a natural and intrinsic way of exploration of search space for multiple knapsack problems (MKP). Their proposed node deployment scheme based on ACO algorithm addressed five deployment scenarios for performance evaluation. The simulations show that the network lifetime can be increased by increasing the energy and density of the sensors closer to the sink. Also it was claimed that this deployment scheme can perform better than other existing schemes and can prolong the network lifetime significantly in any WSN scenario.

The problem of minimum cost and connectivity guaranteed grid coverage (MCGC) is one of the most critical issues for the implementation of WSNs. In [88], a novel algorithm, ant colony optimization with three classes of ant transitions (ACO-TCAT) is proposed to solve this problem. The goal of the algorithm is to improve the quality of the solution space and raise the searching speed. Simulation results are conducted to demonstrate the effectiveness of the proposed approach and they showed better performance than other algorithms like EasiDesign [86] (discussed before). Average steps by an ant for an iteration in ACO-TCAT is much less than that in [86]. It is because in ACO-TCAT, three classes of ant transitions are applied to lessen the candidate points and redundant steps as soon as possible.

#### 4.2.3. ACO in Energy Efficient Clustering

There are a number of works covering the area of energy efficient clustering in WSNs using ACO. Salehpour *et al.* proposed an efficient routing algorithm for the cluster-based large scale WSNs using the ant colony optimization [89]. The technique uses two routing levels: intra-cluster and inter-cluster. In the first level cluster members send data directly to their cluster head. In the second level, the cluster heads use ACO to find a route to the base station. As only cluster heads participate in the inter-cluster routing operation, the method can provide a smooth operation more effectively. As a result this method leads to a shorter convergence time and less routing overhead. To assess the efficiency of the proposed method it was compared with two other algorithms: a cluster-based routing without optimization [58] and an ACO-based routing algorithm without clustering. The results show lower power consumption and more load balancing for the proposed algorithm.

In [90], the authors present a new energy aware clustering protocol, Ant Colony Optimization for Clustering (ACO-C). Using appropriate cost functions (at the base station), the protocol is said to minimize and distribute the cost of long distance transmissions and data aggregation among all sensor nodes evenly. The ACO-C protocol was successfully compared with other well known clustering algorithms like LEACH, LEACH-C and PSO-C over both network lifetime and data delivery to the base station. Their future work will deal with multi-hop routing schemes to improve the lifetime of the network.

In order to improve the energy efficiency and achieve the network load balance, a novel energy efficient unequal clustering scheme for large scale WSNs is proposed in [91]. On the one hand, an improved fuzzy unequal clustering routing (IFUC) algorithm is used to determine one node's chance of becoming cluster head and estimate the cluster-head radius. On the other hand, the ACO is used in energy aware routing between cluster heads (CHs) and base station (BS). It reduces the energy consumption of CHs and solves the hot spots problem which occurs in multi-hop WSN routing in large scales. The experiment results have indicated that the proposed clustering scheme has more superior performance than other methods such as LEACH [58] and EEUC [92].

Another work where an uneven clustering routing algorithm for WSNs based on ACO was proposed is [93]. It utilized the dynamic adaptability and optimization capabilities of the ACO to get the optimum route between the CH. Clusters closer to BS had smaller sizes than those far away from the BS, thus the closer CHs could preserve energy for the inter-cluster data forwarding. Simulation result indicates that the algorithm significantly improved in average energy consumption and survival rate, and extended the network lift cycle compared to LEACH.

#### 4.2.4. ACO in Data Aggregation

A centralized approach to data gathering and communication for WSNs is presented in [94]. The method clearly partitions the work for the BS and sensor nodes according to their different functions and capabilities. A near-optimal chain named AntChain is achieved by using an ACO method running in the BS. The algorithm significantly simplifies the work of sensor nodes. It lowers the communication and computation workload. The authors claim that the AntChain algorithm out-performs LEACH and PEGASIS by eight times and two times, respectively.

In 2006, Misra *et al.* introduced an ant aggregation algorithm for optimal data aggregation in WSNs [95]. They observed that aggregation energy efficiency depends on the number of sources. The results of simulation reveal that optimal aggregation saves energy up to 45% and 20%, respectively, for moderate numbers of source nodes and large numbers of source nodes.

Another scheme for solving the maximum lifetime data gathering problem in distributed intelligent robot networks (DIRNs), as a kind of wireless sensor and actuator network (WSAN), supporting multimedia traffic is developed in [96]. The previous methods used for multimedia traffic provided ineffective exploitation of network resources. With this scheme network lifetime is maximized by jointly optimizing data aggregation variables based on ACO algorithm using bionic swarm intelligence. Furthermore, experimental results demonstrate that the proposed methods attain significant improvements (24% better) in system lifetime compared to other conventional methods such as Minimum Energy Gathering Algorithm (MEGA).

As we have mentioned several times before, that one of the biggest problems of WSN is energy. A few solutions exist to the problem such as LEACH and PEGASIS protocols. While LEACH selects CHs in random manner, the PEGASIS protocol forms a chain of all the nodes in the network, each node taking rounds in transmitting to the BS. In [97] authors discussed about an energy efficient protocol which can enhance the performance of LEACH and PEGASIS. As the nodes are deployed randomly in the area and the BS is located at a distance from them, it is clear that the nodes would actually dissipate energy during their transmission to the BS. The inter-nodal distance also plays a role in unequal energy dissipation of the nodes. This energy difference keeps on increasing resulting in poor network performance. In this scheme, authors claim to nullify the differences occurring due to these above mentioned causes. ACO is used for chain construction instead of the greedy algorithm to enhance the network performance. Extensive simulations have been carried out which showed significant improvement over other schemes.

A new cluster formation technique named energy-efficient data gathering algorithm (EDGA) is discussed in [98], which integrates the advantages of hierarchical routing, chain, and multi-hop routing. A node, according to the degree of support from neighbors and its residual energy, makes its decision to compete for becoming a CH independently. Later, the CH adapts ACO to schedule access sequence of nodes (chain). Simulation results show that EDGA provides lower energy consumption and longer network lifetime than that of conventional algorithms. Xie *et al.* have designed three dynamic ACO based algorithms: SinkDistComb, ResidualEnergy, and SinkAggreDist with improved heuristic rules and node selection rules integrated with in-network data aggregation [99]. They refined the heuristic function and the aggregation node selection method to maximize energy efficiency and to extend network lifetime. Two proposed algorithms are shown to yield longer maximum lifetime and another algorithm is shown to have improved scalability than the conventional algorithms.

### 4.3. Genetic Algorithm

Genetic algorithm (GA) is an evolutionary algorithm which is based on the abstraction of Darwin's evolution of biological systems, pioneered by Holland and his colleagues in the 1960s and 1970s [100]. GA is a particular class of evolutionary algorithm which is categorized as a global search heuristic. GA uses random search in the decision space via selection, crossover and mutation operators in order to reach its goal and attempts to obtain a possibly global optimum answer. Another operator of GA is *elitism.* Its job is to store the best or elite chromosomes (with best fitness values) for the next generation. Genetic algorithms are implemented and presented using simulations. Here population is the abstract representation known as chromosomes and the candidate solutions are known as individuals or phenotypes. Later these are transformed into an optimization problem.

**Figure 7.**
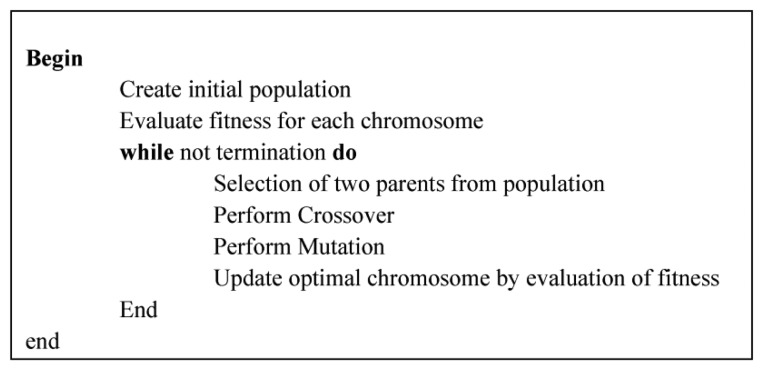
A simple procedure of Genetic Algorithm

GA has mainly two advantages over traditional algorithms which are: the capability of handling complex problems and parallelism. GA can deal with all sorts of objective functions whether they are stationary or transient, linear or nonlinear, continuous or discontinuous. Multiple genes can be suitable for parallel implementation. In GA, basically, the solutions are coded and quantized as binary strings consisting of 0's and 1's. From a population of randomly generated individuals the evolution initiates. In each successive generation, the fitness of every individual in the population is evaluated. From this, multiple individuals are stochastically selected according to their fitness and they form a new population by possible combinations and mutations. The new population is then used in the next generation. The genetic algorithm is summarized in Figure 7. The stopping criteria of GA could be either a predefined number of iterations or convergence during a predefined number of iterations.

#### 4.3.1. GA in WSN Clustering

In WSN clustering, the total energy consumption is closely related with the number of cluster heads and their positions, so it is important to find out an energy-efficient clustering technique that can optimize the energy consumption which is directly related to network lifetime. The first works of clustering in WSNs using genetic algorithms can be found in [101]. Jin *et al.* have used a GA-based method to minimize communication distance in sensor networks via clustering. In their work, GA was used in formation of a number of pre-defined clusters which helped in reducing the total minimum communication distance. The cluster heads (CHs) were adjusted based on fitness function. The algorithm was able to find an appropriate number of cluster-heads and their locations. Results indicate that the number of CHs is about 10% of the total number of nodes. The pre-defined cluster formation also decreased the communication distance by 80% as compared with the distance of direct transmission. In one hand it was able to quickly find good solutions; on the other hand, this algorithm is applicable to both uniform and non-uniform network topologies.

In [102] Hussain *et al.* proposed a genetic algorithm (GA) that was used to create energy efficient clusters for routing in WSNs. In their work, the sink node performed the simultaneous optimization of total dissipated transmission energy and delay by clustering the nodes. The simulation results show that the proposed energy-efficient hierarchical clustering protocol performs better than the traditional cluster- based protocols. The gradual energy depletion in sensor nodes is also investigated.

In another work Hussain *et al.* [103] improved their idea proposed in [102] by improving the fitness function used for GA. This fitness function is based on parameters like cluster size, energy consumption, number of clusters, direct distance to sink *etc*. They extended their work using GA to obtain the optimum number of clusters, CHs, cluster members and transmission schedule. It was shown that their updated method conserves relatively more energy than the method proposed by Jin *et al.* in [101]. They also compared their proposed method with the LEACH protocol in different layouts along with increased number of nodes in the sensor network.

The results of energy saving approaches can vary significantly depending on the number and size of clusters and the distance among the sensor nodes. The authors in [104] aim to find an optimal cluster formation by applying a GA based method in which the chromosome contains the information about the relative position of the nodes. They proposed a location-aware two-dimensional genetic algorithm (LA2D-GA) that performs more efficient in gene evolution than general approach with one-dimensional genetic algorithm (1D-GA). It gives unique location information to each node (chromosome). Thus, when crossover and mutation operations are performed, the optimal clusters can be searched effectively by using this information. The simulation results indicate better performance against LEACH and 1D-GA.

In [105] the authors examine the GA as a dynamic technique to find optimum states. As a simple framework it proposes a mathematical formula, which increases coverage against lifetime. This technique makes a tradeoff between energy consumption and distance parameter. Finally, the proposed algorithm performs better than some traditional cluster-based protocols.

In [106] GA is used for dynamic clustering which is similar to the works we have discussed in [102] and [103]. They used slightly different parameters such as residual energy of the nodes, required energy to send a message toward the sink node, and number of cluster heads. In order to evaluate the algorithm, they simulated the protocol and compared it to LEACH protocol. The simulation results show the effectiveness of the proposed mechanism.

An optimal method of clustering homogeneous WSNs using a multi-objective two-nested GA (M2NGA) is presented in [107]. The network is assumed to be static. The GA is implemented in two levels. In the top level a multi-objective genetic algorithm is used whose goal is to obtain optimum network lifetime for different delay values. In the low level, GA is used for multi hop intra-cluster data transmission, which is not possible in most heuristic clustering methods. The advantage of M2NGA compared with LEACH and other GA based heuristic methods like two tiered GA, is its generality. It is shown that the proposed algorithm yields more efficient clustering schemes in networks in which transmission energy is considerably greater than energy consumed in the electronic circuitry.

#### 4.3.2. GA in WSN Deployment

Due to the energy and other resource constrains in WSNs, activating all the nodes deployed to cover a particular area is not efficient, so activating only the necessary number of nodes at any instance is an efficient way to save the overall energy of the system. To eradicate this problem and extend the network lifetime, a novel searching algorithm, Energy-efficient Coverage Control Algorithm (ECCA), inspired by the multi-objective genetic algorithms (MOGAs [108]), is proposed in [109]. The ECCA have a number of advantages, including very less computation time and one-time resetting of the working state of the sensor nodes. Simulation results showed that the algorithm achieved balanced performance with the same number of deployed sensors on indifferent types of detection sensor models while maintaining high coverage rates.

In [110] Konstantinidis *et al.* investigated the multi-objective deterministic pre-Deployment and Power Assignment Problem (DPAP). DPAP is typical in applications which invoke a limited number of expensive sensors, where their operation is significantly affected by their position and communication [111]. The main motivation of their work was to provide a set of high quality solutions for the DPAP without any prior knowledge on the objectives preference. A multi-objective Evolutionary Algorithm based on Decomposition (MOEA/D) is designed and showed its superiority against MOGA [108] in terms of quality of solutions and convergence speed.

Another GA based multi-objective methodology was implemented for a self-organizing wireless sensor network in [112]. The authors demonstrated the use of GA based node placement methodology for a WSN. The fitness function of the method was designed with taking in account the parameters such as network density, connectivity and energy consumption. In a word they tried to incorporate the network characteristics and application specific requirements in the performance measure of the GA. Along with clustering schemes and transmission signal strengths; GA optimizes the operational modes of the sensor nodes. Dynamic application of the method in WSN layout design can lead to the extension of the network's life span, while keeping the application- specific properties of the network close to the optimal values.

A proper node deployment scheme can reduce the complexity of several parameters in WSNs such as routing, data fusion, communication, *etc.* In [113], Poe *et al.* proposed and investigated random and deterministic node deployments for large-scale WSNs. The performance metrics for the evaluation were: coverage, energy consumption, and message transfer delay. They have considered three competitors: a uniform random, a square grid, and a pattern-based Tri-Hexagon Tiling (THT) node deployment. A simple energy model is formulated to study energy consumption for each deployment strategy. Among the three, THT overall outperforms the other two for energy consumption and worst-case delay. On the other hand the square grid strategy is better than others for coverage performance. They also compared the random deployment strategy with the popular square grid deployment for the worst-case delay.

#### 4.3.3. GA in WSN Routing

Energy efficient routing in WSN is also another area where GA has been implemented. The primary works can be found in [114]. Rahmani *et al.* proposed a parallel GA method in WSN routing. The method aims to find an energy efficient data routing scheme in sensor networks. Simulation results show that the proposed scheme has improved the load balancing and traffic spreading over the network, through the usage of proposed scheme with optimum parameters.

In some WSNs, high energy sensors called relay nodes may form a network among themselves to route data towards the BS. Higher power relay nodes can be used as cluster heads in two-tiered sensor networks to achieve improved network lifetime. In the work [115], the lifetime of a network is determined mainly by the lifetimes of these relay nodes. In this paper, authors proposed a solution, based on a genetic algorithm (GA) for scheduling the data gathering of relay nodes. The proposed algorithm quickly converges to the optimal solution for smaller networks. However, unlike routing formulations based on integer linear program (ILP) [116], the current approach is efficient and is capable of handling much larger networks. Experimental results demonstrated that, compared to other traditional routing schemes (without considering energy dissipation of the nodes), the approach can significantly extend the lifetime of the network by nearly 200% on average.

In [117] the authors considered a two-tiered wireless sensor network, with n relay nodes acting as cluster heads and BS (sink). The assumption was taken that each sensor node belongs to exactly one cluster and the routing schedule is calculated by BS (not power constrained). Sensor nodes transmit their data directly to their respective relay nodes (CH). The relay nodes then perform the initial fusion of the received data and send them to the BS by the routing tree. In order to optimize QoS parameters (delay and reliability) and energy consumptions of WSN, the BS determines a routing tree accordingly based on the residual energy of the node, requested delay and reliability. The proposed protocol reduces average power consumption of nodes and in effect extends the lifetime of the network.

An algorithm called a Quantum Genetic Algorithm (QGA)-based QoS Routing Protocol for WSNs was proposed in [118]. In this paper, they proposed a QoS-based protocol for wireless sensor networks, which can run efficiently with best effort traffic. QGA-QoS is the first quantum genetic algorithm-based QoS routing protocol in wireless sensor networks. QGA can balance between exploration and exploitation easily and effectively. In [119] the authors presented an updated survey and comparative study of some genetic algorithm-based multicast routing techniques. Localization, mobility, query based, energy efficiency, data aggregation and QoS are the metrics used for the genetic algorithm-based multicast routing in wireless sensor networks classification.

In [120], a genetic algorithm-based routing scheme called Genetic Algorithm-based Routing (GAR) is presented that considers the energy consumption issues by minimizing the total distance travelled by the data in every round. Based on the current network state, this GA-based approach can quickly compute a new routing schedule. The computational efficiency of GA to quickly find a solution to the problem is utilized here. The experimental results demonstrate that the proposed algorithm is better than the existing techniques in terms of network life time, energy consumption, and the total distance covered in each round. The experimental results of the simulation showed that it outperforms the Minimum Hop Routing Protocol (MHRM) [121] algorithm by extending the network life time by approximately 230% in contrast to 200% as reported in the GA-based algorithm [115]. However, the algorithm lacks the consideration of residual energy of the relay nodes for energy efficiency.

A new method of clustering (CRCWSN) to transmit data from general nodes to CH and from CH to BS in sensor networks was presented in [122]. The algorithm is based on genetics and re-clustering. These CHs (selected by GA) have been used individually in each round to transmit data. Considering distance and energy parameters, authors have created a target function which has more optimum conditions, compared to previous techniques. Results showed that, at the end of each round, the number of survived (alive) nodes increases, compared to previous methods, resulting in increased network lifetime.

#### 4.3.4. GA in WSN Data Aggregation

As we discussed previously, the fundamental challenge in the design of WSNs is to maximize their lifetimes especially when they have a limited energy supply, so a good data aggregation scheme can change the scenario immensely. In [123] the authors present a genetic algorithm-based approach to generate balanced and energy efficient data aggregation spanning trees for WSNs. In the algorithm, the gene index determines a node and the gene's value identifies the parent node. In the data gathering round, a single best tree consumes the lowest energy among all nodes but assigns more load to selected sensors. The chromosome fitness is determined by four factors: residual energy, transmission, received load, and the distribution of load. Results showed that proposed GA outperforms a few other data aggregation tree-based approaches in terms of extending network lifetime.

In [124], Al Karaki *et al.* presented Grid-based Routing and Aggregator Selection Scheme (GRASS). GRASS is said to provide good solutions for the data gathering and routing problem with in-network aggregation in WSNs with a focus on the joint problem of optimal data routing with data aggregation. They claim that the method can achieve low energy dissipation and low latency without sacrificing quality. GRASS embodies optimal approaches as well as heuristic approaches like Clustering-Based Aggregation Heuristic (CBAH). These algorithms are used to find the minimum number of aggregation points while routing data to the BS. When compared to other schemes, GRASS improves system lifetime with acceptable levels of latency in data aggregation without sacrificing data quality. With 100 nodes CBAH provides almost two times better performance then PEGASIS and almost 1.5 times better performance then LEACH when aggregation is used. Without aggregation the performance of CBAH slightly decreases. Results also demonstrate that the CBAH can increase the system lifetime of large WSNs.

Commonly, the data aggregation tree concept is used to find an energy efficient solution and is largely accepted by the researchers in this area, but fair load sharing is missing in most of these works. To address this issue Norouzi *et al.* [125] presented a method that utilizes genetic algorithm to find routes which balance energy and data load in a network. In this study, nodes monitor the area to aggregate data and then remove the redundant nodes in order to aggregate them according to the data aggregation spanning tree. GA is used here to create an efficient data aggregation tree in which any node has a value property. Like some other methods, the fitness function is determined on the basis of residual energy, number of transmission, and received data packets from individual nodes. The technique is suitable for a homogeneous WSN environment monitoring. Simulation results indicated that this method practically increases the network lifetime compared to other works [112].

### 4.4. Hybrid Approaches

Hybrid approaches are also becoming popular nowadays. In [126], the authors propose a hybrid of PSO and GA for optimization in TDMA scheduling. The performance of this method is compared with PSO, max-degree-first coloring algorithm, and node-based scheduling algorithm. The results show that hybrid algorithm is marginally better (644 mJ of energy) than the schedules determined by max-degree-first coloring algorithm and node-based scheduling algorithm, which consume 740 mJ and 666 mJ, respectively. Moreover, the proposed method can easily make tradeoffs between the time and energy objectives by a proper weight factor.

Another hybrid approach was presented in [127] called LEACH-GA, which has basically the same set-up and steady-state phases of LEACH for each round, with the addition of a preparation phase. In preparation phase optimization is done by GA genetic algorithm-based adaptive clustering protocol with an optimal probability prediction to achieve good performance in terms of lifetime of network in wireless sensor networks. The preparation phase is performed only once before the set-up phase of the first round. This LEACH-GA hybrid method showed almost 40% better lifetime compared to LEACH, almost 400% better lifetime compared to minimum transmission energy (MTE), and nearly 600% better lifetime compared to direct transmission (DT).

### 4.5. Problem Specific Comparison of Existing Bio-Mimic Strategies

**Figure 8.**
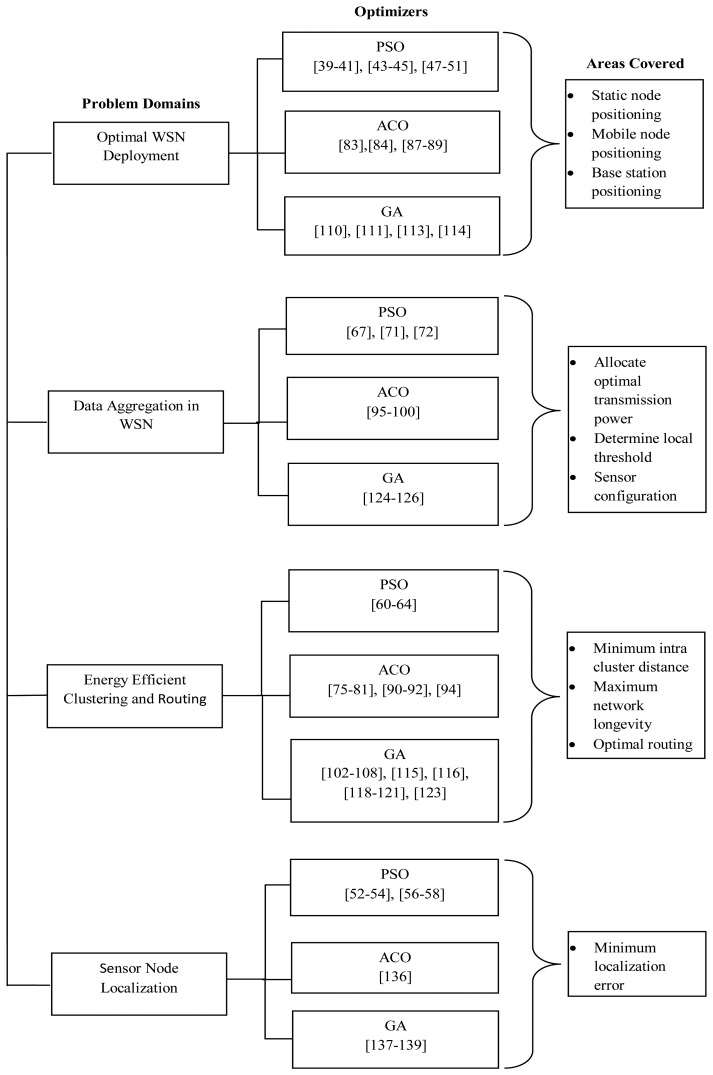
Summary of PSO, ACO and GA based optimizers in WSNs.

From the above study, it is clear that researchers so far tried to implement bio-mimic optimization strategies in a number of problem domains of WSNs. Every approach addressed in this work attempted to solve a specific problem with their own specific set of parameter configurations, their own set of rules, and claimed to show better results with regard to some previous traditional approaches. Also some researchers used hybrid strategies to solve a single problem. But to our knowledge, there is no extensive work, which addresses the comparative study between two or more bio-mimic strategies to solve WSN related problems, so a comparison between these strategies in problem specific view is not a trivial task. In Figure 8, we summarize PSO, ACO, and GA based optimizers that are used in WSNs and their addressed areas. Table 3 summarizes the key features of the aforementioned bio-mimic optimization techniques. Finally, based on the above study and the summary in Table 3, we have summarized our conjecture on problem specific comparison of bio-mimic optimization strategies in Table 4.

## 5. Open Research Issues and Future Directions

**Figure 9.**
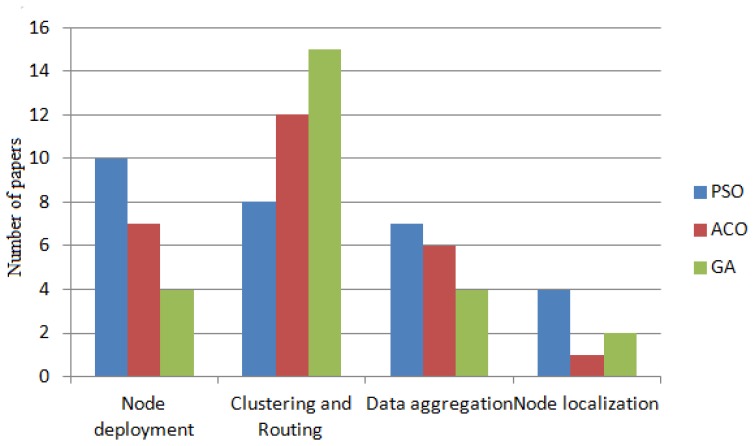
A representation of number of papers published addressing optimization problems in WSN using bio-mimetic methods (non-exhaustive).

Key findings of the study have been summarized in Table 5. In summarizing, characteristics such as node positioning, node localization, data aggregation, clustering, *etc.* are considered. The issues are shown as addressed or not addressed. Figure 9 presents the total number of research works considered in this survey (non-exhaustive list) in recent years covering the optimization techniques in WSN.

Although the bio-mimetic optimization techniques presented herein address many issues of WSNs (Table 5) such as design and deployment, optimal routing and clustering, localization, security, data aggregation, and QoS management, there are still some open research challenges. In particular, research is needed in the area of integration of energy efficiency, QoS, and security. In addition, most previous works view optimization in WSN from a single perspective only. Hence, research in this area addressing the coexistence of all three key issues is limited.

- *Integration of QoS, Energy Efficiency, and Security*: Although the presented approaches address many issues associated with optimization in WSNs, some research questions remain relatively unexplored, such as support for and integration of QoS, energy efficiency and security. In number of WSNs applications such as Body Area Networks, Vehicular *ad hoc* Networks, *etc.* integration of QoS and security along with energy efficiency will be necessary. So integration of these issues in WSNs using metaheuristic algorithms could be a potential future direction.
- *Cross-layer Design*: Generally, issues considered to be optimized are supported by different layers of the network protocol stack of a WSN. For instance, energy efficiency is an issue that needs to be at every layer of the protocol stack, so instead of a strict layered approach, a cross-layer design is desirable. As an example, incorporation of resource awareness in compression schemes, for example dependency on remaining energy, requires coordination between application layer compression and the physical layer. Very little work has been done in cross layer based optimization [128,129]. Exploration of this aspect of compression in WSNs is necessary.
- *Novel Bio-mimetic Algorithms*: Bio mimetic algorithms which have been recently developed could be better alternatives to the existing algorithms. The Firefly Algorithm (FA) is such a novel algorithm which was developed by Xin-She Yang in 2007 [132,133]. It is based on the flashing patterns and behavior of fireflies. A discrete version of FA can efficiently solve NP-hard scheduling problems [132], while a detailed analysis has demonstrated the efficiency of FA over a wide range of test problems, including multi objective load dispatch problems [133].
- Cuckoo search (CS) is one of the latest nature-inspired metaheuristic algorithms, developed in 2009 by Yang and Deb [28]. CS is based on the brood parasitism of some cuckoo species. In addition, this algorithm is enhanced by a special type of randomization named “L'evy flights” [134], rather than by simple isotropic random walks. Recent studies show that CS is potentially far more efficient than PSO and GAs [135]. Bat-inspired algorithm is another novel bio-mimic optimization algorithm developed by Yang in 2010. This bat algorithm is based on the echolocation behavior of micro bats with varying pulse rates of emission and loudness [29]. So the consideration of these novel approaches in WSNs for optimization purposes can be a future direction.
- *Real Implementation*: From the above study it is found that many bio-mimetic optimization methods have outperformed conventional methods under certain environments. However, most existing bio-mimetic optimization works are simulation-based and only a a few have been evaluated in real WSN environments. Implementation of these methods in real WSN environments or test-beds could be a fruitful future research direction.
- *Placement of Implementation*: The implementation of most existing bio-mimic algorithms are in base station or sink (centralized), which need communication between the nodes. This communication can be very frequent and expensive in dynamic WSNs environment. Distributed implementation of these algorithms in lightweight form could be a potential future direction.

## 6. Conclusion and Future Work

Development of effective optimization algorithms is the key to improve the utilization of the limited resources of WSNs (energy, bandwidth, computational power). A large number of diverse bio-mimetic algorithms have addressed issues such as design and deployment, localization, security, energy efficient routing and clustering, scheduling, data aggregation, and fusion *etc.* In this work, we have made an effort to put these works into perspective and to present a holistic view of the field. In addition, a general review of current state-of-the-art is presented along with their advantages and limitations, which can be served as a future guide for using bio-mimetic algorithms for WSNs.

In doing this, we have provided a comprehensive overview of the three main existing approaches, namely PSO, ACO, and GA. Each category has a number of variants, which are presented accordingly. Some hybrid approaches and few novel bio-inspired approaches are also discussed as future research directions. Cross-layer design and parameter learning in optimization is envisioned to be another interesting new research area in WSNs. Most issues arise from cross-layer incompatibility and high physical intervention is needed for parameter setting and adjustment, so more sophisticated learning platforms and paradigms are necessary rather than specific solutions.

Although the presented approaches address many issues associated with optimization in WSNs, some research questions remain relatively unexplored, such as support for and integration of QoS, energy efficiency and security. There is significant amount of scope for future work in these areas. Realizing the importance of these issues in WSNs, our future endeavors will focus on developing a framework which integrates QoS-awareness, energy efficiency and security for WSNs. The diverse applications of WSNs demand support for a diverse set of QoS requirements. A single technique will not be optimal for all applications. Along with the abovementioned points, a secondary objective will be to determine the best possible technique for a particular application taking into account the limited available resources. We also have the intention to explore the possibilities of cross-layer design approaches in WSNs.

## Figures and Tables

**Table 1.** Notations used in PSO.

*w*	inertia weight
$v_{id}^{n}$	old velocity calculated for each particle
$v_{id}^{n+1}$	new velocity calculated for each particle
*c*_1_ and *c*_2_	self confidence factor and the swarm confidence factor
*r*_1_ and *r*_2_	random numbers
$p_{id}^{n}$	particles own past best position
$x_{id}^{n}$	old position calculated for each particle
$p_{gd}^{n}$	the best position a particle attained in the whole swarm

**Table 2.** Summary of PSO approaches in WSNs.

**Authors**	**Literature**	**Main Contributions**	**Area of Optimization**
Aziz *et al.*	Particle Swarm Optimization and Voronoi diagram for Wireless Sensor Networks coverage optimization [40].	Minimize the area of coverage holes Finds close to optimal coverage Uses centralized PSO-Voronoi algorithm	Stationary Node Deployment

Hu *et al.*	Topology optimization for urban traffic sensor network [41]	Real world traffic surveillance Uses binary PSO Minimization of cost of sensor equipment	Stationary Node Deployment

Ngatchou *et al.*	Distributed sensor placement with sequential particle swarm optimization [43]	Maritime surveillance application Uses a sequential form of PSO	Stationary Node Deployment

Li *et al.*	Improving sensing coverage of wireless sensor networks by employing mobile robots [44]	Improve the QoS in sensing coverage Uses particle swarm genetic optimization (PSGO)	Hybrid Deployment

Wang *et al.*	An improved co-evolutionary particle swarm optimization for wireless sensor networks with dynamic deployment [45]	Competent for dynamic deployment in WSNs and has better performance and efficiency Uses virtual force directed co-evolutionary particle swarm optimization (VFCPSO)	Dynamic Deployment

Hong *et al.*	Allocating multiple base stations under general power consumption by the particle swarm optimization [47]	Finds multiple base stations Assures maximum network life	Base Station Positioning

Mendis *et al.*	Optimized sink node path using particle swarm optimization [48]	Target is to achieve the optimal path for sink node Good approach for sparse deployment	Base Station Positioning

Nascimento *et al.*	A Particle Swarm Optimization Based Approach for the Maximum Coverage Problem in Cellular Base Stations Positioning [49]	Focuses on the trade-off between the total Capital Expenditure to implement the network and Quality of Service (QoS) PSO algorithm determines the placement of the BS	Base Station Positioning

Gopakumar *et al*	Localization in wireless sensor networks using particle swarm optimization [52]	Minimize localization error Performs better than simulated annealing	Node Localization

Kulkarni *et al.*	Bio-inspired node localization in wireless sensor networks [54]	Uses bacterial foraging algorithm along with PSO focused on range-based distributed iterative node localization	Node Localization

Low *et al.*	A particle swarm optimization approach for the localization of a wireless sensor network [55]	PSO-based distributed localization scheme No beacons Good performance as compared with the Gauss- Newton algorithm (GNA)	Node Localization

Low *et al.*	Optimization of sensor node locations in a wireless sensor network [56]	A localization scheme for unknown emitter nodes To obtain better estimated location of the sensor nodes PSO is used	Node Localization

Tillet *et al.*	Cluster-head identification in ad hoc sensor networks using particle swarm optimization [58]	Uses PSO to equalize the number of nodes and candidate CH in each cluster Minimizes the energy spent by the nodes and maximizes the data transmission	Energy Aware Clustering

Latiff *et al.*	Energy-aware clustering for wireless sensor networks using particle swarm optimization [59]	Defined a new cost function Proposed protocol selects a high-energy node as a CH and produces clusters that are equally placed throughout the entire WSN field	Energy Aware Clustering

Chunlin *et al.*	Particle swarm optimization for mobile ad hoc networks clustering [60]	Divided Range Particle Swarm Optimization (DRFSO) algorithm was applied to the revised Weighted Clustering Algorithm flexibility of assigning different weights to the nodes	Energy Aware Clustering

Guru *et al.*	Particle swarm optimizers for cluster formation in wireless sensor networks [61]	Four variants of PSO were proposed Considers only the physical distances between nodes and their assigned cluster-heads	Energy Aware Clustering

Cao *et al.*	Cluster heads election analysis for multi-hop wireless sensor networks based on weighted graph and particle swarm optimization [62]	Node and its CH is engaged in a multi-hop communication CHs were elected by maximum residual energy and in turns and by probabilities separately	Energy Aware Clustering

Wimalajeewa *et al.*	Optimal power scheduling for correlated data fusion in wireless sensor networks via constrained PSO [66]	Addresses the problem of optimal power allocation through constrained PSO Objective is to minimize the energy expenditure while keeping the fusion-error probability under a required threshold	Data Aggregation

Veeramachaneni *et al.*	Swarm intelligence based optimization and control of decentralized serial sensor networks [67]	Hybrid approach of ant-based control and PSO for hierarchy and threshold management 40% performance improvements in terms of Bayesian risk value	Data Aggregation

Veeramachaneni *et al.*	Dynamic sensor management using multi objective particle swarm optimizer [68]	A binary multi objective PSO for optimal sensor management PSO is modified to optimize two objectives: accuracy and time	Data Aggregation

Guo *et al.*	Multi-Source Temporal Data Aggregation in Wireless Sensor Networks [70]	A multi-source temporal data aggregation model is presented Proposes an energy-efficient multi-source temporal data aggregation model called MSTDA	Data Aggregation

Jiang *et al.*	Linear Decision Fusion under the Control of Constrained PSO for WSNs [71]	Designed a linear decision fusion rule Proposed a way of controlling the parameters of the model taking the advantage of the constrained PSO	Data Aggregation

**Table 3.** Advantages and disadvantages of major bio-mimetic optimization algorithms.

**Algorithm Name**	**Advantages**	**Disadvantages**
PSO	- Easy to implement - Few parameters to adjust - Efficient in global search	- Iterative nature can prohibit it's use for high-speed real-time applications - If optimization needs to be carried out frequently it's not that convenient - Requires large amounts of memory, which may limit its implementation to resource constraint base stations - Easily drops into regional optimum or local minima

ACO	- Inherent parallelism - Can be used in dynamic applications - Positive Feedback leads to rapid discovery of good solutions - Distributed computation avoids premature convergence	- Theoretical analysis is difficult - Probability distribution changes in every iteration - Convergence is guaranteed, but time to convergence uncertain - Coding is not straightforward

GA	- It can solve every optimization problem which can be described with the chromosome encoding - GA is not dependent on the error surface, so we can solve multi-dimensional, non-differential, non-continuous, and even non-parametrical problems. - Genetic algorithms are easily transferred to existing simulations and models	- Longer running times - It cannot assure constant optimization response times - It cannot handle a population with a lot of subjects

**Table 4.** Strengths of major bio-mimetic optimization algorithms in solving WSN problems.

**Optimization Strategies ▸**		**PSO**	**ACO**	**GA**
Problem Domains	Optimal WSN Deployment	Centralized nature of PSO minimizes the area of coverage holes of stationary node positioning.	Distributed nature of ACO is better in solving mobile node deployment issues.	Good for random as well as for deterministic node deployments.

	Data Aggregation in WSN	Data aggregation is a repetitive process which is quite suitable for PSO.	In case of large scale and dynamic WSNs it can perform better.	Suitable in finding minimum number of aggregation points while routing data to the BS.

	Energy Efficient Clustering and Routing	PSO shows better performance in selecting the high energy node as CHs in each round and can find optimal route effectively.	Performs better in maximizing both network lifetime and data delivery to the base station.	GA is used in formation of a number of pre-defined clusters, which helped in reducing the overall minimum communication distance.

	Sensor Node Localization	Minimizes the localization error effectively	Improves the accuracy of the unknown node location.	Global searching capability of GA obtains better estimated location of the sensor nodes.

**Table 5.** A summary of major bio-mimetic optimization methods in WSNs.

**Optimization Methods ▸** **Areas Covered** **(Issues addressed) ▾**	**PSO**	**ACO**	**GA**
Stationary Node Deployment (QoS)	Addressed	Addressed	Addressed
Hybrid Deployment (QoS)	Addressed	Not addressed	Addressed
Dynamic Deployment (QoS)	Addressed	Not Addressed	Addressed
Base Station Positioning	Addressed	Not addressed	Not addressed
Node Localization (EE)	Addressed	Addressed	Addressed
Energy Aware Clustering (EE)	Addressed	Addressed	Addressed
Data Aggregation and fusion (Data Security)	Addressed	Addressed	Addressed
Cross Layer Optimization	Addressed	Addressed	Not addressed
Optimal Routing	Addressed	Addressed	Addressed
